# Factors associated with work-life balance among emergency physicians, nurses, and paramedics: a systematic review

**DOI:** 10.3389/fpubh.2026.1830270

**Published:** 2026-05-25

**Authors:** Bartosz Pryba, Beata Wieczorek-Wójcik, Katarzyna Pietrzak, Aleksandra Gaworska-Krzemińska

**Affiliations:** 1 Independent Monoprofile Medical Simulation Laboratory, Institute of Nursing and Midwifery, Faculty of Health Sciences with the Institute of Maritime and Tropical Medicine, Medical University of Gdańsk, Gdańsk, Poland; 2 Division on Nursing Management, Institute of Nursing and Midwifery, Faculty of Health Sciences with the Institute of Maritime and Tropical Medicine, Medical University of Gdańsk, Gdańsk, Poland

**Keywords:** emergency medical technicians, emergency nursing, family conflict, physicians, work-life balance, workload

## Abstract

**Introduction:**

This mixed-methods systematic review aimed to identify and synthetically evaluate factors associated with work-life balance (WLB) among emergency physicians, emergency nurses, and paramedics.

**Methods:**

The review was conducted in accordance with the PRISMA 2020 guidelines and was prospectively registered in the PROSPERO database. A comprehensive search of PubMed, Scopus, Web of Science, and CINAHL was performed, encompassing English-language publications from 2015 to 2025. Inclusion criteria were defined using the PICOS framework. Methodological quality was appraised using Joanna Briggs Institute (JBI) critical appraisal tools. Furthermore, the overall certainty and confidence in the evidence were assessed using the GRADE (for quantitative data) and GRADE-CERQual (for qualitative data) frameworks.

**Results:**

From 201 identified records, 10 studies met the inclusion criteria: nine quantitative cross-sectional studies and one qualitative study. The findings indicate that the primary determinants of WLB impairment among emergency personnel include high occupational stress, Effort-Reward Imbalance (ERI), Work–Family Conflict (WFC), shift-work patterns, and adverse organizational characteristics, such as staffing shortages and limited team support. Numerous studies demonstrated robust associations between WLB disturbances and professional burnout, deterioration of somatic and mental health, sleep disorders, and diminished job satisfaction. According to GRADE criteria, the certainty of quantitative evidence was predominantly rated as low or very low; however, the qualitative study provided evidence of moderate-to-high confidence (GRADE-CERQual).

**Discussion:**

The findings underscore the critical necessity of implementing multilevel organizational interventions aimed at enhancing working conditions, mitigating work–family conflict (WFC), and bolstering team-based support. Such strategies are pivotal for safeguarding the occupational well-being of the emergency medicine workforce. Addressing these structural determinants is essential to ensure long-term staff retention and the sustainability of emergency care systems.

**Systematic review registration:**

https://www.crd.york.ac.uk/PROSPERO/view/CRD420251237774, identifier: CRD420251237774.

## 1 Introduction

Emergency Medical Services (EMS) represent a fundamental component of modern healthcare systems, operating at the intersection of acute care, public health, and social safety. Emergency physicians, nurses, and paramedics are routinely exposed to high-intensity workloads, severe time pressure, unpredictable clinical scenarios, and profound emotional burdens—including exposure to severe trauma, sudden death, and the care of highly vulnerable patient populations. Consequently, emergency personnel constitute an occupational group at elevated risk for work-related stress, burnout, and impaired work-life balance (WLB). These challenges have significant implications not only for the individual well-being of healthcare professionals but also for workforce retention and the overall quality of patient care (1–5).

Work-life balance (WLB) is a multidimensional construct reflecting the subjectively perceived ability to reconcile professional demands with personal, family, and social life. In the healthcare sector, impaired WLB is consistently associated with adverse psychological and somatic outcomes, including emotional exhaustion, depersonalization, sleep disturbances, somatic complaints, and depressive symptoms (3, 4, 6–9). From a public health perspective, diminished WLB among emergency medical personnel also translates to increased sickness absence, higher turnover intention, decreased job satisfaction, and weakened organizational commitment. Consequently, these factors exacerbate workforce shortages within already overburdened emergency systems (3, 10–12).

A growing body of evidence indicates that WLB in emergency medicine is determined not solely by individual characteristics, but primarily by structural and organizational factors. High workloads, staffing shortages, shift work schedules, substantial administrative burdens, effort-reward imbalance (ERI) are among the most frequently identified determinants of occupational stress across diverse healthcare systems and cultural contexts. These are compounded by bidirectional work-family/family-work conflicts (WFC/FWC) treated as conceptually distinct constructs, reflecting different directions of interference between work and family domains (6, 7, 12–18). Furthermore, recent evidence suggests that the impact of ERI on health outcomes may be indirect, mediated by emotional exhaustion and bidirectional work–family conflicts (WFC/FWC). This underscores the complex psychosocial mechanisms linking the work environment to employee well-being (7, 12–16). Modifying factors - such as gender, career stage, and professional role - also play a crucial role; notably, younger clinicians and female professionals more frequently report poorer WLB, elevated burnout levels, and higher turnover intentions (1, 2, 8, 12, 19–22).

Despite a growing body of empirical research, the available evidence remains fragmented, methodologically heterogeneous, and of variable quality. The majority of publications rely on cross-sectional designs, which precludes causal inference, while the relative impact of specific occupational factors across distinct emergency medicine professions has not yet been adequately systematized. Furthermore, quantitative findings are rarely integrated with qualitative data, which are crucial for capturing the subjective experiences of personnel and the phenomenon of work-to-life stress spillover (11, 23–26).

Given this context, there is a compelling need for a systematic review grounded in rigorous methodological standards. The primary aim of this study is to consolidate the current state of knowledge, identify consistent determinants of WLB among emergency physicians, nurses, and paramedics, and critically appraise the quality and certainty of the available evidence. Although existing systematic reviews have predominantly investigated burnout and WLB within isolated professional cohorts, they offer limited insight into cross-professional dynamics. This siloed approach to evaluating nurses, paramedics, and emergency physicians creates a critical gap in the comparative synthesis of WLB determinants across the multidisciplinary emergency care workforce (27–30). To achieve this, we conducted a mixed-methods evidence synthesis, utilizing recognized frameworks for methodological appraisal and certainty grading. Although the included literature was predominantly quantitative, this integrated approach was adopted to capture the contextual and experiential depth provided by the single qualitative study. Consequently, the qualitative data were utilized as an explanatory mechanism to complement and interpret the quantitative results, rather than to generate independent or comparative conclusions (31–34).

This mixed-methods systematic review was guided by the following research questions:

Q1: What are the main organizational and system-level factors (e.g., shift work organization and workforce shortages) that determine work-life balance (WLB) among frontline physicians, nurses, and paramedics?

Q2: How do work–family conflict (WFC) and effort–reward imbalance (ERI) mediate the relationship between occupational stress and adverse health outcomes (such as emotional exhaustion) in this specific professional group?

Q3: How do experiences of organizational friction and the resulting stress-coping strategies differ according to the specific professional roles operating within the same high-risk emergency care environment.

## 2 Methods

### 2.1 Study design

The mixed-methods systematic review was performed between November 2025 and December 2025 in accordance with the Preferred Reporting Items for Systematic Reviews and Meta-Analyses (PRISMA) 2020 guidelines (31). The systematic review protocol was registered in the PROSPERO database (ID 1237774). Studies meeting the inclusion criteria were categorized into quantitative and qualitative designs. The Risk of Bias was assessed using the appropriate Joanna Briggs Institute (JBI) Critical Appraisal Checklists (32). Subsequently, the overall certainty of evidence was evaluated using the GRADE framework for quantitative findings and the GRADE-CERQual approach for qualitative evidence (33, 34).

### 2.2 Search strategy

To systematically guide the search strategy, research questions were formulated according to the PICOS framework (34). This structured approach facilitated the identification of core keywords and the definition of inclusion and exclusion criteria (Table 1.).

**Table 1:** Inclusion and exclusion criteria for the systematic review (PICOS) (34).

PICOS framework	Inclusion criteria	Exclusion criteria
P (population)	Emergency nurses, emergency physicians, paramedics	Healthcare professionals working outside of emergency settings (e.g., intensive care unit [ICU] staff, general ward nurses) or mixed clinical cohorts where data for emergency personnel cannot be extracted separately
I (intervention/ exposure)	Workplace environmental and organizational factors (e.g., workload, shift work impact, organizational support)	Focus on non-occupational stressors or personal lifestyle interventions unrelated to the organizational work environment
C (comparison)	Different levels of workplace exposures, or not applicable	Not applicable
O (outcome)	Work-life balance (WLB) and its related indicators, including work–family conflict (WFC), family–work conflict (FWC), occupational burnout, and job satisfaction	Studies reporting exclusively on clinical patient outcomes (e.g., quality of care, patient safety) or strictly biomedical indicators (e.g., fertility, reproductive health) without evaluating WLB, occupational stress, or burnout
S (type of study)	Quantitative (experimental, randomized controlled, longitudinal, cross-sectional) and qualitative studies	Secondary research (systematic reviews, meta-analyses), non-empirical literature (editorials, opinion pieces, letters to the editor, reports), and single case reports

A comprehensive literature search was conducted across the following electronic databases: PubMed, Scopus, Web of Science, and CINAHL (via EBSCOhost). The search strategy utilized the following keywords: “Emergency Nurs*,” “Emergency Physicians,” “Paramedics,” “Work Environment,” “Occupational Stress,” “Organizational Culture,” “Work-life Balance,” and “Family Conflict.” These terms were combined into search strings using Boolean operators (AND, OR), as detailed. The search results were subsequently restricted using specific filters:

- language: English,
- full-text available,
- publication year: 2015–2025.

The initial database search yielded 200 articles. An additional relevant record was identified through other sources (e.g., citation searching), resulting in a total of 201 articles for the initial screening phase.

### 2.3 Data extraction

**Figure 1:**
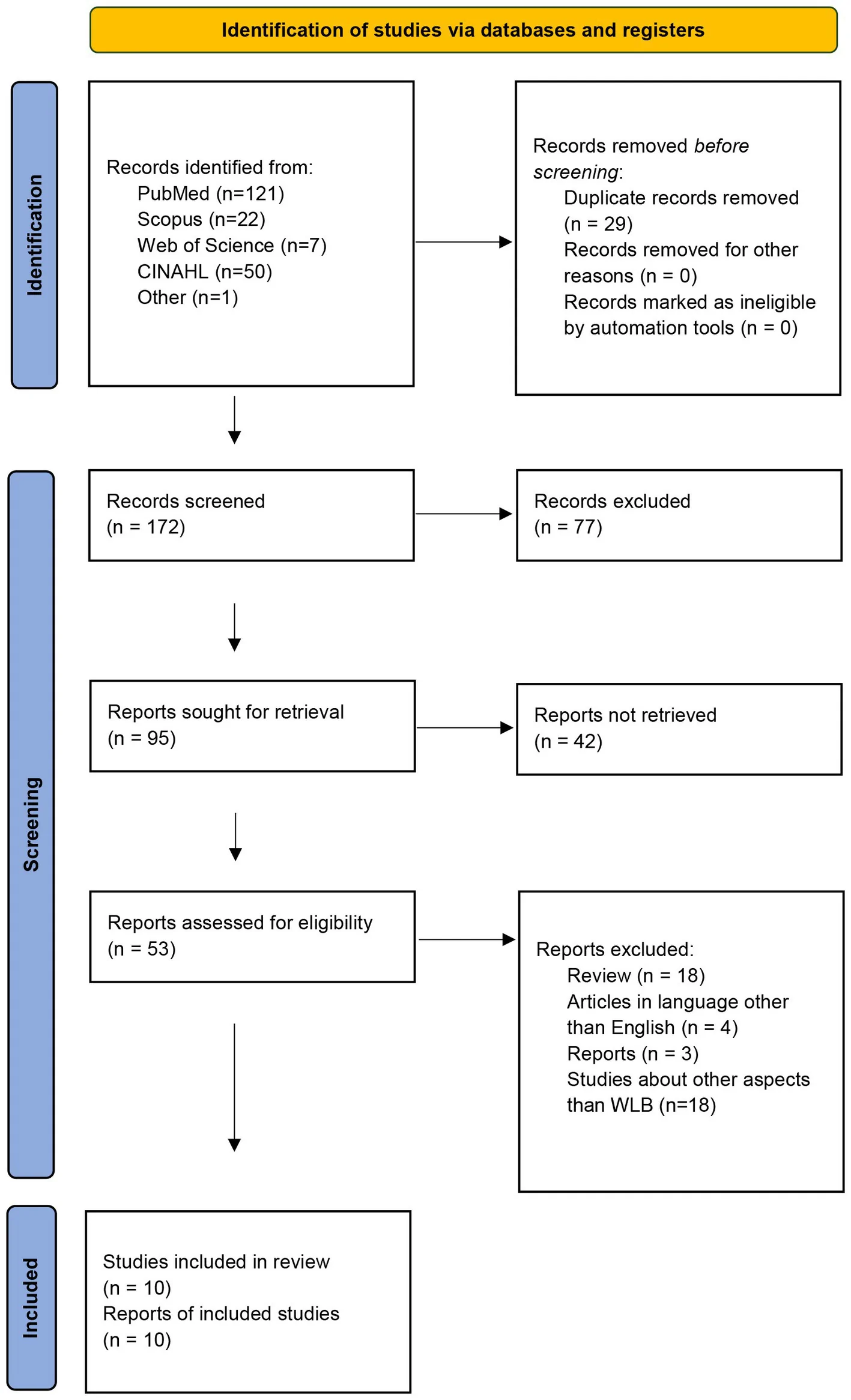
Prisma flow diagram (31).

Article screening was performed by three independent reviewers using the Rayyan tool, in accordance with the PRISMA 2020 guidelines. Data were extracted independently by the authors, capturing the following: author and year of publication, country, study design, study population, sample size, measurement tools, analyzed exposures, main outcomes regarding WLB/WFC/burnout/occupational stress, and key methodological limitations. Discrepancies were resolved through discussion and consensus. Ultimately, 10 out of 201 studies were included in the final analysis, comprising nine quantitative studies and one qualitative study. The complete study selection process is illustrated in the PRISMA flow diagram (Figure 1). As all quantitative studies utilized a cross-sectional design, the Risk of Bias within these studies was assessed using the 8-item JBI Critical Appraisal Checklist for Analytical Cross Sectional Studies (Supplementary Table S1). The qualitative study was evaluated using the 10-item JBI Critical Appraisal Checklist for Qualitative Research (Table 2). For both JBI tools, items were scored as “Yes,” “No,” “Unclear,” or “Not applicable.” Finally, the overall certainty of evidence was evaluated using the Grading of Recommendations Assessment, Development and Evaluation (GRADE) approach for quantitative findings (Supplementary Table S2) and the Confidence in the Evidence from Reviews of Qualitative Research (GRADE-CERQual) approach for qualitative data (Table 3).

Due to the substantial heterogeneity across study populations, measurement instruments, definitions of WLB/WFC, and reported outcomes, a statistical meta-analysis was not feasible. Instead, a narrative synthesis approach was employed. The findings were grouped according to the main categories of identified factors: occupational burden, effort-reward imbalance, work-family conflict, shift work organization, organizational support, and subsequent health and professional consequences.

**Table 2:** Methodological quality assessment of the included qualitative study using the JBI Critical Appraisal Checklist (32).

No.	JBI critical appraisal checklist for qualitative research criteria	First author, Year of Publication: Osmančević et al. (23)
1.	Is there congruity between the stated philosophical perspective and the research methodology?	Yes
2.	Is there congruity between the research methodology and the research question or objectives?	Yes
3.	Is there congruity between the research methodology and the methods used to collect data?	Yes
4.	Is there congruity between the research methodology and the representation and analysis of data?	Yes
5.	Is there congruity between the research methodology and the interpretation of results?	Yes
6.	Is there a statement locating the researcher culturally or theoretically?	Unclear
7.	Is the influence of the researcher on the research, and vice- versa, addressed?	No
8.	Are participants, and their voices, adequately represented?	Yes
9.	Is the research ethical according to current criteria or, for recent studies, and is there evidence of ethical approval by an appropriate body?	Yes
10.	Do the conclusions drawn in the research report flow from the analysis, or interpretation, of the data?	Yes

**Table 3:** Assessment of confidence in the evidence from the included qualitative study using the GRADE-CERQual approach (33).

No.	GRADE-CERQual criteria	First author, Year of Publication: Osmančević et al. (23)	Concerns level
1.	Methodological limitations	Methodological rigor was supported by a descriptive-interpretive design, clear sampling criteria, ethical approval, and investigator triangulation during data analysis. However, the absence of a reflexivity statement regarding researcher positionality constitutes a notable methodological gap	Moderate concerns
2.	Coherence	High coherence: Strong alignment between the raw empirical data and the identified themes. The rigorous application of inductive thematic analysis facilitated the extraction of internally consistent constructs, which are thoroughly substantiated by participant verbatim quotes	No concerns
3.	Adequacy of data	No concerns regarding data adequacy. The findings are grounded in a robust sample size for qualitative research (*n* = 26 interviews), ensuring high data saturation. Furthermore, the interdisciplinary nature of the study population provides a valuable multi-perspectivity of data, culminating in a rich, thick description of the studied phenomenon	No concerns
4.	Relevance	Minor concerns regarding relevance. While the findings are highly applicable to the review’s objective, transferability may be slightly limited by the single-center design and the specific sociocultural and geographical context of the study setting (Istria, Croatia)	Minor concerns
Confidence Assessment		Moderate confidence. It is likely that the review finding is a reasonable representation of the phenomenon of interest. The study provides a trustworthy account of how working conditions impact the well-being of emergency personnel, though minor concerns regarding transferability and reflexivity prevent a ‘High’ rating	

## 3 Results

The initial search strategy yielded a total of 201 records, including 200 records retrieved from the across selected databases (PubMed: 121, Cinahl: 50, Scopus: 22, Web of Science: 7) and one additional record identified through other sources. After removing 29 duplicate records, 173 unique citations underwent title and abstract screening, leading to the exclusion of 119 irrelevant records. A thorough full-text assessment for eligibility was performed on the remaining 54 articles. Ultimately, 10 studies (nine quantitative and one qualitative) met all inclusion criteria and were included in the final synthesis.

Synthesizing the Risk of Bias assessment conducted via the JBI Critical Appraisal Checklist, the quantitative literature demonstrates a notable strength in the employment of validated measurement scales. However, a recurrent limitation across these studies is the omission of essential multivariate analyses required to rigorously control for confounding variables. Conversely, the included qualitative study Osmančević et al. (23) exhibits a sound methodological framework, yielding well-documented and logically derived conclusions from the data. Its primary methodological deficit, however, is the absence of a researcher reflexivity statement - specifically, the failure to delineate the power dynamics and the relationship between the researchers and participants, which may have compromised the candor of the respondents. As all included quantitative studies utilized non-randomized, observational designs, their initial certainty of evidence was graded as Low. Following a comprehensive GRADE assessment, the majority of the studies (n = 6) were downgraded to Very Low certainty. This reduction was primarily driven by a high risk of bias and the absence of multivariate statistical analyses to control for confounding variables; additionally, severe imprecision due to small sample sizes contributed to the downgrade in three of these cases. Three studies (n = 3) maintained their initial Low certainty rating without further downgrades.

According to the GRADE-CERQual approach, confidence in the findings of the single qualitative study (n = 1) was rated as Moderate. The initial high rating was downgraded due to notable methodological deficits, specifically the absence of a reflexivity statement and the risk of insider bias, which the original authors failed to critically deconstruct within their manuscript. Furthermore, the overall confidence rating was slightly penalized by minor limitations in the transferability of the results, inherent to the study’s single-center design and its specific geographical and cultural context.

The studies included in this systematic review explored critical dimensions of work-life balance (WLB) among emergency physicians, emergency nurses, and paramedics. The prevalence rates and determinants of occupational burnout—specifically stress and effort were examined in five studies reward Imbalance (ERI)—were examined in five studies (*n* = 5) (1, 2, 6, 16, 35). The impact of work–family conflict (WFC) on overall WLB and subsequent health deterioration was investigated in two studies (*n* = 2) (7, 16). Furthermore, four studies (*n* = 4) analyzed specific workplace environmental factors (7, 11, 17, 23), while also four studies (*n* = 4) highlighted variations in burnout and work-life imbalance across different socio-demographic groups (1, 2, 8, 35). A detailed description of the elements included is provided in Supplementary Table S3.

In the context of emergency medicine, work–family conflict (WFC) frequently serves as a significant mediator and moderator in the relationship between occupational stress (such as effort–reward imbalance) and health outcomes, including emotional exhaustion and somatic symptoms (7, 16). The multidisciplinary emergency care team—comprising physicians, nurses, and paramedics—endures a severe psychophysical toll from shift work, clinically manifesting as chronic sleep disorders, clinical depression, and multi-organ somatization (1, 7, 11, 16). Moreover, the synthesized literature delineates a robust sociodemographic trend: female professionals, unmarried individuals, and early-career clinicians (e.g., residents) bear a disproportionate risk of severe psychophysical exhaustion (1, 2, 35). Despite distinct clinical scopes of practice, fundamental systemic deficits—such as inadequate institutional support, suboptimal teamwork culture, and erratic scheduling—persistently devastate the wellbeing of the entire workforce (1, 2, 35).

Evaluating the mechanisms of burnout demonstrates that pathological work environments strike clinical professions in varied ways, yet constitute an inextricably linked system of burdens. Among physicians, especially those undergoing residency training, cognitive overload and an absence of rostering autonomy act as critical stressors, driving the highest prevalence of full syndrome burnout (2, 35). Among the nursing workforce, this pathomechanism is fueled by an objective effort–reward imbalance (ERI). The stark asymmetry between extreme clinical engagement and the lack of commensurate reward generates severe emotional exhaustion, acting as the primary mediator for sleep disorders (7, 16). For EMS personnel, the dominant stressors are physical and behavioral. Extreme ergonomic burdens, alongside chronic disruptions to biological rhythms and nutritional habits—characterized by “opportunistic eating”—drive up sickness absence rates and drastically erode their occupational quality of life (11, 23). The unifying element across these professional cohorts is an environmental vicious cycle: the physical depletion of paramedics, the emotional exhaustion of nurses, and the cognitive overload of physicians converge during shared clinical interventions. Consequently, the ensuing degradation of communication and the erosion of teamwork exponentially amplify occupational stress and turnover intention among all constituents of the emergency care system (11, 16, 23).

## 4 Discussion

This systematic review synthesized current evidence on the factors associated with work-life balance (WLB) among emergency physicians, emergency nurses, and paramedics. Our findings demonstrate that WLB disruptions within these occupational groups are driven primarily by systemic and organizational factors, with individual characteristics playing only a secondary role. Across the analyzed literature, three interrelated mechanisms emerged most strongly and consistently: effort-reward imbalance (ERI), work–family conflict (WFC), and the substantial occupational burden stemming from shift work organization and chronic staffing shortages (6, 7, 16, 17). Of particular significance is the finding that work–family conflict (WFC) functions not merely as a co-occurring phenomenon, but actively serves as a mediating variable between effort–reward imbalance and adverse health outcomes—such as somatic symptoms and sleep disturbances—specifically among emergency nurses (7, 16). This relational pattern supports the interpretation of WLB as an indicator highly sensitive to the “work architecture” of high-risk environments, rather than merely a reflection of individual characteristics. Our findings heavily align with the international literature on the burnout and well-being of healthcare professionals, which emphasizes the superiority of organizational determinants—such as strategic staffing, professional autonomy, supervisor support, and a culture of psychological safety—over interventions focusing exclusively on individual resilience (e.g., mindfulness training) (29, 36, 37), a conclusion further corroborated by recent reviews confirming demanding work environments as the primary antecedents of burnout across all emergency department roles (38).

Recent meta-analyses demonstrate that although both individual coping strategies and organizational redesign mitigate burnout, systemic interventions remain indispensable for securing long-term, population-level efficacy within the healthcare workforce (5, 36). This distinction is of paramount importance in the context of emergency medicine. While the sheer clinical workload, extreme time pressures, and the inherent unpredictability of critical events constitute largely unmodifiable occupational hazards, the strategic organization of shift schedules, staffing adequacy, and institutional support represent highly actionable avenues for organizational intervention. While organizational factors critically shape WLB, a comprehensive understanding of this phenomenon requires examining individual coping strategies and resilience. Research indicates that clinicians navigate clinical pressure by integrating problem-focused strategies (e.g., task prioritization) with emotion-focused techniques, such as brief mental resets and emotional distancing (39). While adaptive behaviors like positive reframing and peer support build resilience, chronic overload often triggers maladaptive responses, including emotional venting or substance use (40). Furthermore, resilience is mediated not only by personal competencies but also by family capital and supportive workplace leadership (41). Crucially, professional identity—an integral component of WLB—is severely degraded by moral injury (42). The necessity to deliver substandard care due to severe staffing shortages violates ethical frameworks, compelling personnel to establish strict protective boundaries, such as reducing clinical hours or leaving the profession entirely, to preserve their psychological integrity (43).

The systemic failures and psychological burdens described above manifest distinctly across professional roles. Within the domain of emergency nursing, these results strongly align with broader reviews on ED nurse burnout. The prevailing evidence highlights the paramount role of structural factors—namely job demands, decision latitude (job control), and social support—alongside the severe psychological toll of cumulative exposure to traumatic events (29), a burden that is significantly amplified during macro-level health emergencies (44). Similarly, effective leadership and management styles serve as crucial predictors of an employee’s intention to stay (12). Crucially, evidence drawn from general shift-work nursing literature outside the ED strongly correlates extended shifts (≥ 12 h) with exacerbated burnout, reduced job satisfaction, and higher rates of intention to leave the profession (47, 48). Viewed through the lens of WLB, this implies that while individual practitioners frequently favor compressed schedules (≥ 12-h shifts) to secure more leisure days, the overarching health and organizational toll remains negative across the workforce. This is especially true in high-acuity environments characterized by chronic staffing shortages and frequent night shift rotations.

Among paramedics and Emergency Medical Services (EMS) personnel, the mental health burden and the psychological sequelae of cumulative exposure to critical incidents are particularly pronounced. The meta-analysis by Petrie et al. (28) regarding the prevalence of PTSD and common mental disorders within ambulance crews corroborates that this specific workforce faces a disproportionately high occupational risk (49). In a complementary vein, the qualitative review by Lawn et al. (30) examining the wellbeing of ambulance personnel delineates five core thematic domains of occupational impact. These range from profound psychological and psychosocial burdens, through the somatic impact of chronic stress, to the overarching role of organizational culture and structural work design (30). These findings align with the single qualitative study included in our review, which highlighted the “spillover effect” of occupational stress into family life, concomitant sleep disturbances, and the profound difficulty personnel face in separating their professional and personal identities. Furthermore, complementary quantitative data from cohort and cross-sectional designs reinforce the critical role of EMS-specific stressors as primary predictors of adverse health outcomes and diminished job satisfaction (50).

An essential complement to interpreting these results is the lens of professional identity and retention drivers within EMS. As demonstrated by a Finnish study (48), specific dimensions of the professional role are closely linked to turnover intention. This strongly suggests that WLB and occupational well-being should not be evaluated in a vacuum; rather, they must be analyzed concurrently with Person-Job fit - specifically, the congruence between a practitioner’s role expectations and the actual organizational realities.

The complexity of WLB is further amplified by structural variances across global healthcare macrosystems. International workforce mapping (e.g., in Australia, Canada, Rwanda, and South Africa) reveals that structural fragmentation and severe urban–rural maldistribution drastically exacerbate local workloads (49). These macrosystemic differences directly dictate occupational security; for instance, evidence from Portugal demonstrates that secure employment status (e.g., civil service) acts as a critical buffer against emotional exhaustion compared to precarious contracts (50). Consequently, these vulnerabilities create a highly interdependent global medical market. Pervasive burnout and inadequate national policies drive the brain drain phenomenon, accelerating workforce migration from low- to high-income countries (49). Integrating these individual and macrosystemic paradigms provides profound insight into the structural determinants of the global healthcare workforce crisis.

**Figure 2:**
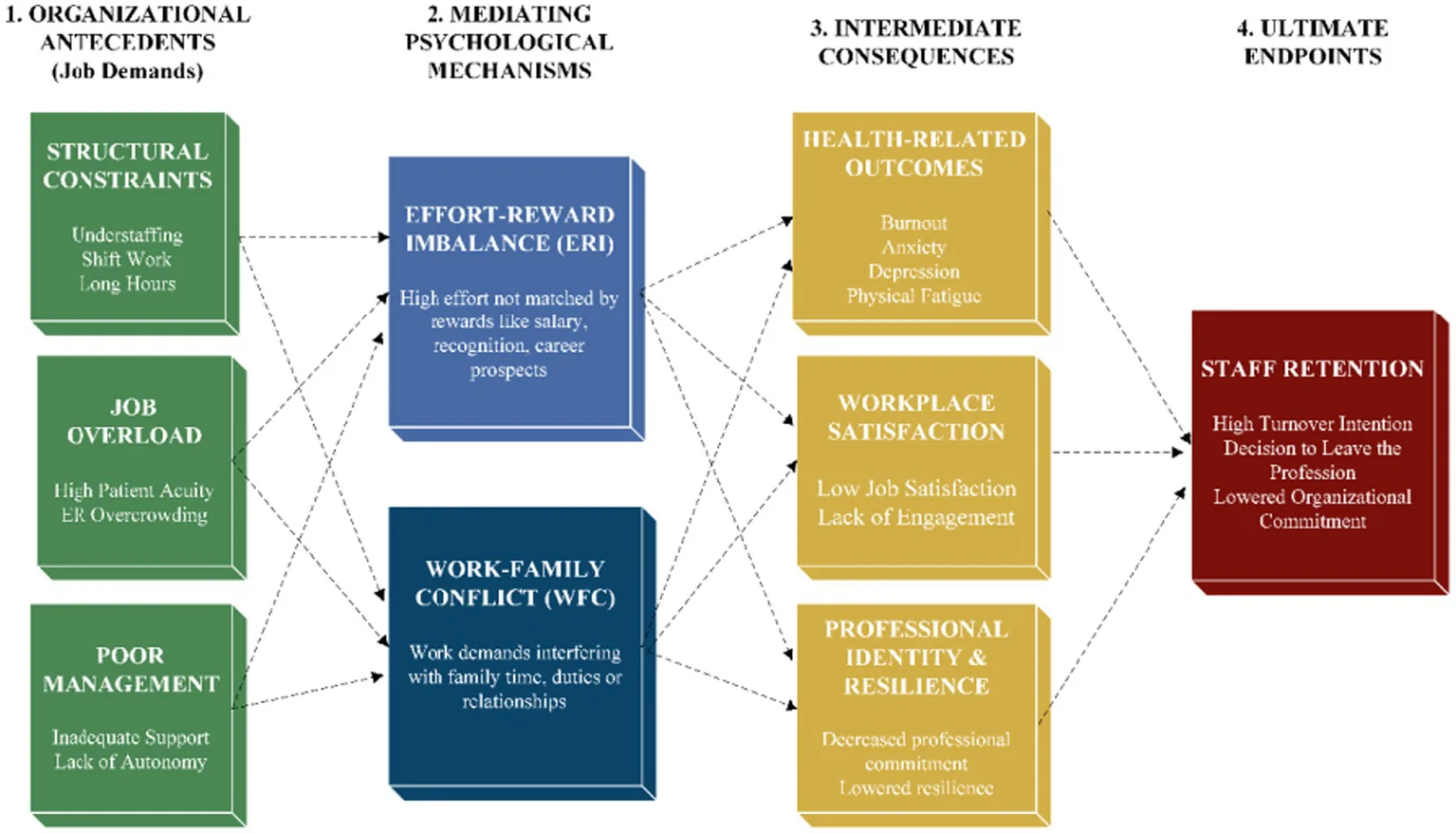
Conceptual framework of work-life balance determinants and outcomes in emergency care settings (51). Dashed arrows indicate hypothesized directional associations based on the cross-sectional evidence synthesized in this review, as causal relationships cannot be definitively established.

Drawing upon our synthesized findings and the overarching Job Demands-Resources (JD-R) model, we propose a conceptual framework (Figure 2) elucidating the relationships among the identified constructs (51). Crucially, adverse organizational conditions - such as chronic understaffing and demanding shift work - do not drive turnover intention directly. Rather, they operate through two primary mediating mechanisms: precipitating WFC and exacerbating ERI. It is the cumulative burden of these adverse psychological states that precipitates health deterioration, diminishes job satisfaction, and ultimately solidifies turnover intention. Comprehending this mechanistic pathway is vital for healthcare management, as it demonstrates that strategic interventions must not only target structural redesign but also actively buffer ERI and WFC to ensure workforce retention.

### 4.1 Practical implications for health policy

From a practical and policy-oriented perspective, grounded in the overarching JD-R theoretical framework, our findings suggest potential avenues for a multilevel intervention model (51). To buffer job demands and enhance resources, organizational strategies might focus on mitigating Effort-Reward Imbalance (ERI) by instituting highly transparent and equitable frameworks for remuneration, professional recognition, and career advancement (e.g., standardized salary matrices, peer-nominated recognition programs and career development pathways). Concurrently, decisive structural actions are required to curtail the ‘compensatory overexertion’ chronically forced upon frontline staff by severe workforce shortages perationally achievable through the strict enforcement of safe patient-to-staff ratios (7, 16, 52, 53). Secondly, targeted interventions are imperative to effectively mitigate Work–Family Conflict (WFC). At the operational level, this necessitates optimizing the frequency of night shifts (e.g., to a maximum of three), implementing mandatory four-week advance rostering, ensuring genuine autonomy in shift-swapping (e.g., via digital platforms), and providing robust institutional support via childcare subsidies or on-site care facilities (54–57). Thirdly, the literature concerning organizational strategies for medical staff well-being (37) underscores the paramount importance of leadership quality, organizational culture, and systemic interventions, such as standardized interprofessional triage algorithms and dedicated leadership training programs. This framework is especially critical within ED and EMS environments, where ‘organizational friction’ - manifesting as staffing deficits, patient flow bottlenecks, and role conflict - rapidly cascades into severe psychological overload (58).

### 4.2 Limitations of evidence and future research directions

Restricting the inclusion criteria solely to full-text, English-language publications introduces an inherent risk of selection bias - specifically, language bias. This methodological constraint potentially omits pertinent literature published in other languages, thereby limiting the generalizability of our findings across diverse cultural and structural healthcare contexts. A further critical limitation across the synthesized body of evidence is the overwhelming predominance of cross-sectional study designs, precluding robust causal inference and the evaluation of the longitudinal impact of organizational interventions on WLB, but also introduces a substantial risk of reverse causality—for instance, personnel already experiencing high baseline stress may perceive their work-family boundaries more negatively. Furthermore, the quantitative corpus is hindered by substantial methodological heterogeneity regarding measurement instruments (e.g., varying definitions of WLB/WFC, diverse scales for burnout and somatic symptoms). This pervasive lack of standardization precluded the feasibility of a quantitative meta-analysis. Additionally, the primary studies show a high susceptibility to selection bias driven by low response rates and reliance on convenience sampling. Consequently, the overall certainty of the quantitative evidence was downgraded to low - a finding consistent with broader systematic reviews evaluating clinician well-being. Conversely, the integrated qualitative data provided a crucial explanatory mechanism, contextualizing exactly how the structural burdens of emergency care permeate the private sphere, hese findings are inherently context-bound and may lack broader transferability (23, 30). Given the predominance of cross-sectional study designs and the generally low certainty of evidence (GRADE) across the included literature, the recommendations outlined in our Practical Implications for Health Policy section cannot establish definitive causality. Consequently, the proposed interventions should be approached cautiously as exploratory strategies, requiring continuous, prospective evaluation prior to widespread generalizability. Future research must prioritize longitudinal designs and the rigorous standardization of WLB and WFC measures specifically tailored to emergency care settings. Furthermore, there is a critical need to evaluate the efficacy of organizational interventions using quasi-experimental frameworks (e.g., pre-post designs following roster or staffing modifications). Such studies must unequivocally incorporate objective endpoints - including absenteeism, staff turnover, and medical error rates—alongside tangible patient outcomes and the overall quality of care.

## 5 Conclusion

In conclusion, while the reliance on cross-sectional designs yields a low certainty of evidence regarding direct causality, this systematic review indicates that Work-Life Balance (WLB) impairment within emergency medicine is fundamentally a consequence of adverse working conditions—specifically, Effort-Reward Imbalance (ERI), Work–Family Conflict (WFC), and organizational factors inherent to shift work and chronic understaffing (7, 16, 17, 28–30, 45, 46) Consequently, despite these methodological constraints, enhancing WLB must be recognized as a critical public health and workforce policy objective. Achieving this requires robust systemic interventions, moving definitively beyond strategies that merely target individual ‘resilience’.

## Data Availability

The original contributions presented in the study are included in the article/Supplementary material, further inquiries can be directed to the corresponding author.
