# Correction: Factors associated with work-life balance among emergency physicians, nurses, and paramedics: a systematic review

**DOI:** 10.3389/fpubh.2026.1951408

**Published:** 2026-08-27

**Authors:** Bartosz Pryba, Beata Wieczorek-Wójcik, Katarzyna Pietrzak, Aleksandra Gaworska-Krzemińska

**Affiliations:** 1 Independent Monoprofile Medical Simulation Laboratory, Institute of Nursing and Midwifery, Faculty of Health Sciences with the Institute of Maritime and Tropical Medicine, Medical University of Gdańsk, Gdańsk, Poland; 2 Division on Nursing Management, Institute of Nursing and Midwifery, Faculty of Health Sciences with the Institute of Maritime and Tropical Medicine, Medical University of Gdańsk, Gdańsk, Poland

**Keywords:** emergency medical technicians, emergency nursing, family conflict, physicians, work-life balance, workload

In the **abstract**, the review methodology was generally classified as a systematic review, and the number of included studies erroneously accounted for an excluded article. This has been corrected to read:

“**Introduction:** This mixed-methods systematic review aimed to identify and synthetically evaluate factors associated with work-life balance (WLB) among emergency physicians, emergency nurses, and paramedics.”

“**Results:** From 201 identified records, 10 studies met the inclusion criteria: nine quantitative cross-sectional studies and one qualitative study.”

There was a mistake in Figure 1 as published. The diagram inaccurately reported databases, registers, and language criteria, and mistakenly included an excluded study (Estryn-Behar M. et al., 2011). The corrected Figure 1 appears below.

**Figure 1:**
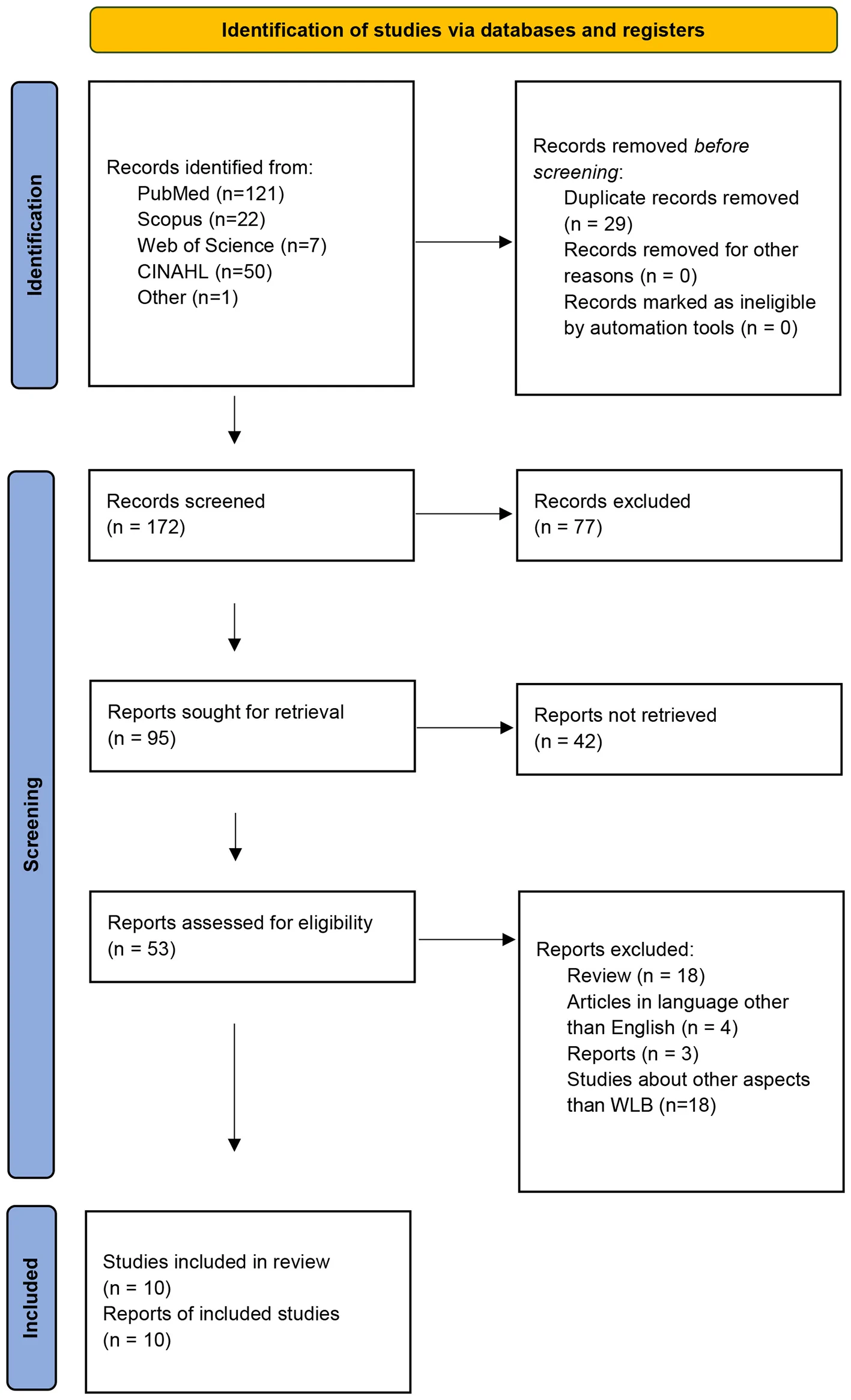
Prisma flow diagram (31).

There was a mistake in **Supplementary Table S1** as published. The table mistakenly included an excluded study (Estryn-Behar M. et al., 2011). The corrected **Supplementary Table S1** has been updated.

There was a mistake in **Supplementary Table S2** as published. The table mistakenly included an excluded study (Estryn-Behar M. et al., 2011). The corrected **Supplementary Table S2** has been updated.

There was a mistake in **Supplementary Table S3** as published. The table mistakenly included an excluded study (Estryn-Behar M. et al., 2011). The corrected **Supplementary Table S3** has been updated.

There was a mistake in the caption of **Supplementary Tables S1, S2** as published. The captions lacked optimal terminological precision regarding the framework utilized. The corrected captions of **Supplementary Tables S1, S2** appears below.

“**Supplementary Table S1**. Risk of Bias assessment of the included cross-sectional studies using the JBI Critical Appraisal Checklist (22).'

“**Supplementary Table S2**. Risk of Bias assessment of the included qualitative study using the JBI Critical Appraisal Checklist (32).”

The **Generative AI statement** was erroneously given as ‘The author(s) declared that Generative AI was not used in the creation of this manuscript'. The correct **Generative AI statement** is: “The author(s) declared that Generative AI was used in the creation of this manuscript. AI-assisted technologies (e.g., translation software and large language models) were utilized strictly for language editing, translation refinement, and structural formatting of the English text to ensure clarity. The authors assume full responsibility for the study design, data extraction, analysis, synthesis, and the final content of the manuscript.”

Reference Williamson et al. [8] was inadvertently omitted from the in-text citations. The citation has now been inserted in the **Section 3**, paragraph 4 and should read:

“The prevalence rates and determinants of occupational burnout—specifically stress and Effort-Reward Imbalance (ERI)—were examined in five studies (*n* = 5) (1, 2, 6, 16, 35). The impact of work–family conflict (WFC) on overall WLB and subsequent health deterioration was investigated in two studies (*n* = 2) (7, 16). Furthermore, four studies (*n* = 4) analyzed specific workplace environmental factors (7, 11, 17, 23), while also four studies (*n* = 4) highlighted variations in burnout and work-life imbalance across different socio-demographic groups (1, 2, 8, 35). A detailed description of the elements included is provided in **Supplementary Table S3**.”

Yang et al. (2025) was not cited in the article. The citation has now been inserted in the **Section 4**, paragraph 1 and should read: This systematic review synthesized current evidence on the factors associated with work-life balance (WLB) among emergency physicians, emergency nurses, and paramedics. Our findings demonstrate that WLB disruptions within these occupational groups are driven primarily by systemic and organizational factors, with individual characteristics playing only a secondary role. Across the analyzed literature, three interrelated mechanisms emerged most strongly and consistently: effort-reward imbalance (ERI), work–family conflict (WFC), and the substantial occupational burden stemming from shift work organization and chronic staffing shortages (6, 7, 16, 17). Of particular significance is the finding that work–family conflict (WFC) functions not merely as a co-occurring phenomenon, but actively serves as a mediating variable between effort–reward imbalance and adverse health outcomes—such as somatic symptoms and sleep disturbances—specifically among emergency nurses (7, 16). This relational pattern supports the interpretation of WLB as an indicator highly sensitive to the “work architecture” of high-risk environments, rather than merely a reflection of individual characteristics. Our findings heavily align with the international literature on the burnout and well-being of healthcare professionals, which emphasizes the superiority of organizational determinants—such as strategic staffing, professional autonomy, supervisor support, and a culture of psychological safety—over interventions focusing exclusively on individual resilience (e.g., mindfulness training) (29, 36, 37), a conclusion further corroborated by recent reviews confirming demanding work environments as the primary antecedents of burnout across all emergency department roles (38).

Liang et al. (2025) was not cited in the article. The citation has now been inserted in the **Section 4**, paragraph 3 and should read: The systemic failures and psychological burdens described above manifest distinctly across professional roles. Within the domain of emergency nursing, these results strongly align with broader reviews on ED nurse burnout. The prevailing evidence highlights the paramount role of structural factors—namely job demands, decision latitude (job control), and social support—alongside the severe psychological toll of cumulative exposure to traumatic events (29), a burden that is significantly amplified during macro-level health emergencies (44). Similarly, effective leadership and management styles serve as crucial predictors of an employee's intention to stay (12). Crucially, evidence drawn from general shift-work nursing literature outside the ED strongly correlates extended shifts (≥12 h) with exacerbated burnout, reduced job satisfaction, and higher rates of intention to leave the profession (45, 46). Viewed through the lens of WLB, this implies that while individual practitioners frequently favor compressed schedules (≥12-h shifts) to secure more leisure days, the overarching health and organizational toll remains negative across the workforce. This is especially true in high-acuity environments characterized by chronic staffing shortages and frequent night shift rotations.

To accurately reflect the convergent mixed-methods design, clarify the Risk of Bias assessment, ensure perfect consistency with the PRISMA guidelines, and exclude an article falling outside the chronological inclusion criteria (Estryn-Behar M. et al., 2011), the methodology, results, and discussion required revisions.

A correction has been made to the **Section 1**, paragraph 6: “This mixed-methods systematic review was guided by the following research questions:

Q1: What are the main organizational and system-level factors (e.g., shift work organization and workforce shortages) that determine work-life balance (WLB) among frontline physicians, nurses, and paramedics?

Q2: How do work–family conflict (WFC) and effort–reward imbalance (ERI) mediate the relationship between occupational stress and adverse health outcomes (such as emotional exhaustion) in this specific professional group?

Q3: How do experiences of organizational friction and the resulting stress-coping strategies differ according to the specific professional roles operating within the same high-risk emergency care environment.”

A correction has been made to the **Section 2**, *Section 2.1*, paragraph 1: “The mixed-methods systematic review was performed between November 2025 and December 2025 in accordance with the Preferred Reporting Items for Systematic Reviews and Meta-Analyses (PRISMA) 2020 guidelines (31). The systematic review protocol was registered in the PROSPERO database (ID 1237774). Studies meeting the inclusion criteria were categorized into quantitative and qualitative designs. The Risk of Bias was assessed using the appropriate Joanna Briggs Institute (JBI) Critical Appraisal Checklists (32).”

A correction has been made to the **Section 2**, *Section 2.2*, paragraph 3: “The initial database search yielded 200 articles. An additional relevant record was identified through other sources (e.g., citation searching), resulting in a total of 201 articles for the initial screening phase.”

A correction has been made to the *Section 2.3*, paragraphs 1 and 2: “Article screening was performed by three independent reviewers using the Rayyan tool, in accordance with the PRISMA 2020 guidelines. Data were extracted independently by the authors, capturing the following: author and year of publication, country, study design, study population, sample size, measurement tools, analyzed exposures, main outcomes regarding WLB/WFC/burnout/occupational stress, and key methodological limitations. Discrepancies were resolved through discussion and consensus. Ultimately, 10 out of 201 studies were included in the final analysis, comprising nine quantitative studies and one qualitative study. The complete study selection process is illustrated in the PRISMA flow diagram (Figure 1). As all quantitative studies utilized a cross-sectional design, the Risk of Bias within these studies was assessed using the 8-item JBI Critical Appraisal Checklist for Analytical Cross Sectional Studies (**Supplementary Table S1**). The qualitative study was evaluated using the 10-item JBI Critical Appraisal Checklist for Qualitative Research (Table 2). For both JBI tools, items were scored as “Yes,” “No,” “Unclear,” or “Not applicable.” Finally, the overall certainty of evidence was evaluated using the Grading of Recommendations Assessment, Development and Evaluation (GRADE) approach for quantitative findings (**Supplementary Table S2**) and the Confidence in the Evidence from Reviews of Qualitative Research (GRADE-CERQual) approach for qualitative data (Table 3).

Due to the substantial heterogeneity across study populations, measurement instruments, definitions of WLB/WFC, and reported outcomes, a statistical meta-analysis was not feasible. Instead, a narrative synthesis approach was employed. The findings were grouped according to the main categories of identified factors: occupational burden, effort-reward imbalance, work-family conflict, shift work organization, organizational support, and subsequent health and professional consequences.”

A correction has been made to the **Section 3**, paragraphs 1 and 2: “The initial search strategy yielded a total of 201 records, including 200 records retrieved from the across selected databases (PubMed: 121, Cinahl: 50, Scopus: 22, Web of Science: 7) and one additional record identified through other sources. After removing 29 duplicate records, 173 unique citations underwent title and abstract screening, leading to the exclusion of 119 irrelevant records. A thorough full-text assessment for eligibility was performed on the remaining 54 articles. Ultimately, 10 studies (nine quantitative and one qualitative) met all inclusion criteria and were included in the final synthesis.

Synthesizing the Risk of Bias assessment conducted via the JBI Critical Appraisal Checklist, the quantitative literature demonstrates a notable strength in the employment of validated measurement scales.”

A correction has been made to the section by removing text **Section 3**, paragraph 2: “Conversely, one study (*n* = 1) was upgraded to Moderate certainty due to a large magnitude of effect (indicated by substantial Odds Ratios) and the presence of a distinct dose-response gradient.”

A correction has been made to the **Section 3**, paragraphs 4–6: “The studies included in this systematic review explored critical dimensions of work-life balance (WLB) among emergency physicians, emergency nurses, and paramedics. The prevalence rates and determinants of occupational burnout—specifically stress and effort were examined in five studies reward Imbalance (ERI)—were examined in five studies (*n* = 5) (1, 2, 6, 16, 35). The impact of work–family conflict (WFC) on overall WLB and subsequent health deterioration was investigated in two studies (*n* = 2) (7, 16). Furthermore, four studies (*n* = 4) analyzed specific workplace environmental factors (7, 11, 17, 23), while also four studies (*n* = 4) highlighted variations in burnout and work-life imbalance across different socio-demographic groups (1, 2, 8, 35). A detailed description of the elements included is provided in **Supplementary Table S3**.

In the context of emergency medicine, work–family conflict (WFC) frequently serves as a significant mediator and moderator in the relationship between occupational stress (such as effort–reward imbalance) and health outcomes, including emotional exhaustion and somatic symptoms (7, 16). The multidisciplinary emergency care team—comprising physicians, nurses, and paramedics—endures a severe psychophysical toll from shift work, clinically manifesting as chronic sleep disorders, clinical depression, and multi-organ somatization (1, 7, 11, 16). Moreover, the synthesized literature delineates a robust sociodemographic trend: female professionals, unmarried individuals, and early-career clinicians (e.g., residents) bear a disproportionate risk of severe psychophysical exhaustion (1, 2, 35). Despite distinct clinical scopes of practice, fundamental systemic deficits—such as inadequate institutional support, suboptimal teamwork culture, and erratic scheduling—persistently devastate the wellbeing of the entire workforce (1, 2, 35).

Evaluating the mechanisms of burnout demonstrates that pathological work environments strike clinical professions in varied ways, yet constitute an inextricably linked system of burdens. Among physicians, especially those undergoing residency training, cognitive overload and an absence of rostering autonomy act as critical stressors, driving the highest prevalence of full-syndrome burnout (2, 35). Among the nursing workforce, this pathomechanism is fueled by an objective effort–reward imbalance (ERI). The stark asymmetry between extreme clinical engagement and the lack of commensurate reward generates severe emotional exhaustion, acting as the primary mediator for sleep disorders (7, 16). For EMS personnel, the dominant stressors are physical and behavioral. Extreme ergonomic burdens, alongside chronic disruptions to biological rhythms and nutritional habits—characterized by “opportunistic eating”—drive up sickness absence rates and drastically erode their occupational quality of life (11, 23). The unifying element across these professional cohorts is an environmental vicious cycle: the physical depletion of paramedics, the emotional exhaustion of nurses, and the cognitive overload of physicians converge during shared clinical interventions. Consequently, the ensuing degradation of communication and the erosion of teamwork exponentially amplify occupational stress and turnover intention among all constituents of the emergency care system (11, 16, 23).”

A correction has been made to the **Section 4**, paragraph 4: “Among paramedics and Emergency Medical Services (EMS) personnel, the mental health burden and the psychological sequelae of cumulative exposure to critical incidents are particularly pronounced. The meta-analysis by Petrie et al. (2018) regarding the prevalence of PTSD and common mental disorders within ambulance crews corroborates that this specific workforce faces a disproportionately high occupational risk (47). In a complementary vein, the qualitative review by Lawn et al. (2020) examining the wellbeing of ambulance personnel delineates five core thematic domains of occupational impact. These range from profound psychological and psychosocial burdens, through the somatic impact of chronic stress, to the overarching role of organizational culture and structural work design (30). These findings align with the single qualitative study included in our review, which highlighted the “spillover effect” of occupational stress into family life, concomitant sleep disturbances, and the profound difficulty personnel face in separating their professional and personal identities. Furthermore, complementary quantitative data from cohort and cross-sectional designs reinforce the critical role of EMS-specific stressors as primary predictors of adverse health outcomes and diminished job satisfaction (48).”

The original version of this article has been updated.

