# Supplementary material for: Factors associated with work-life balance among emergency physicians, nurses, and paramedics: a systematic review
Source: Front Public Health. 2026 May 25;14:1830270. doi: 10.3389/fpubh.2026.1830270 (PMC13244636; doi:10.3389/fpubh.2026.1830270)
Supplement: Supplementary file 2 [file Supplementary_file_1.docx]

Supplementary Material

**Supplementary Table 1.** Risk of Bias assessment of the included cross-sectional studies using the JBI Critical Appraisal Checklist (22).

| No. | First author, Year of Publication | Were the criteria for inclusion in the sample clearly defined? | Were the study subjects and the setting described in detail? | Was the exposure measured in a valid and reliable way? | Were objective, standard criteria used for measurement of the condition? | Were confounding factors identified? | Were strategies to deal with confounding factors stated? | Were the outcomes measured in a valid and reliable way? | Was appropriate statistical analysis used? |
| --- | --- | --- | --- | --- | --- | --- | --- | --- | --- |
| 1. | Skotnicki B. (2024) | Yes | Yes | Yes | Yes | Yes | Yes | Unclear | Yes |
| 2. | Sánchez Onrubia I. (2025) | Yes | Yes | Yes | Yes | Yes | No | Yes | Yes |
| 3. | Nowrouzi-Kia B. (2022) | Yes | Yes | Yes | Yes | Yes | Yes | Yes | Yes |
| 4. | Sonmez L. (2021) | Yes | Yes | Yes | Yes | Yes | No | Yes | Yes |
| 5. | Feeks C. (2020) | Yes | Yes | Yes | Yes | Yes | Yes | Yes | Yes |
| 6. | Song N. (2022) | Yes | Yes | Yes | Yes | Yes | Yes | Yes | Yes |
| 7. | Tong L. (2024) | Yes | Yes | Yes | Yes | Yes | Yes | Yes | Yes |
| 8. | Tan Y. (2025) | Yes | Yes | Yes | Yes | Yes | Yes | Yes | Yes |
| 9. | Williamson K. (2018) | Yes | Yes | Yes | Yes | Yes | No | Yes | Yes |

**Supplementary Table 2.** Risk of Bias assessment of the included qualitative study using the JBI Critical Appraisal Checklist (32).

| No. | Article |  | GRADE criteria |  |  |  |  | Summary |  |
| --- | --- | --- | --- | --- | --- | --- | --- | --- | --- |
|  | First author, Year of Publication | Type of study | Risk of Bias | Inconsistency of results | Indirectness | Imprecision | Publication Bias | Quality of evidence | Comment |
| 1. | Skotnicki B. (2024) | Non-RCT | VS** | *NS** | *NS** | *NS** | ND*** | Very low (**⨁◯◯◯)** | High risk of selection bias and reliance on unvalidated single-item measures |
| 2. | Sánchez Onrubia I. (2025) | Non-RCT | VS** | *NS** | *NS** | VS** | ND*** | Very low (**⨁◯◯◯)** | Downgraded for imprecision (small sample size, n = 74) and high risk of bias (failure to adjust for confounders using only unadjusted bivariate tests) |
| 4. | Nowrouzi-Kia B. (2022) | Non-RCT | VS** | *NS** | *NS** | *NS** | ND*** | Very low (**⨁◯◯◯)** | Serious risk of non-response bias due to a low response rate (22%) |
| 5. | Sonmez L. (2021) | Non-RCT | *VS*** | *NS** | *NS** | *VS*** | ND*** | Very low (**⨁◯◯◯)** | Downgraded for imprecision (n = 141) and high risk of confounding (absence of multivariate regression analysis) |
| 6. | Feeks C. (2020) | Non-RCT | VS** | *NS** | *NS** | VS** | ND*** | Very low (**⨁◯◯◯)** | Serious risk of bias: low response rate (30%) and failure to control for confounding variables |
| 7. | Song N. (2022) | Non-RCT | *NS** | *NS** | *NS** | *NS** | ND*** | Low (**⨁⨁◯◯)** | Large sample size, minimizing the risk of imprecision |
| 8. | Tong L. (2024) | Non-RCT | *NS** | *NS** | *NS** | *NS** | ND*** | Low (**⨁⨁◯◯)** | Robust methodology: large multi-center sample and advanced statistical modeling |
| 9. | Tan Y. (2025) | Non-RCT | *NS** | *NS** | *NS** | *NS** | ND*** | Low (**⨁⨁◯◯)** | Robust methodology: large multi-center sample and advanced statistical modeling |
| 10. | Williamson K. (2018) | Non-RCT | VS** | *NS** | *NS** | *NS** | ND*** | Very low (**⨁◯◯◯)** | Serious risk of bias: failure to control for confounding variables in the correlational analysis |

** NS – No Serious,* 

*** VS – Very Serious*

**** ND – Not Detectected*

**Supplementary Table 3.** Systematic Review of literature presenting factors associated with Work-Life Balance Among Emergency Physicians, Nurses, and Paramedics

| First author, Year of Publication | Aim of study | Type of study | Study group | Methodology | Results | Conclusions | Limitations |
| --- | --- | --- | --- | --- | --- | --- | --- |
| Skotnicki B. (2024) | To assess gender differences in work-life integration, career satisfaction, and burnout among pediatric emergency medicine (PEM) physicians, and to identify associated individual and occupational determinants | A cross-sectional observational study | Pediatric emergency medicine physicians (N=239) | The questionnaire underwent content validation via expert review, literature synthesis, focus groups, and a pilot study (think-aloud protocol). Work-Life Integration (WLI) and career satisfaction were assessed using single-item measures rated on a 7-point Likert scale. Burnout (emotional exhaustion, depersonalization) was evaluated using a validated 2-item abbreviation of the Maslach Burnout Inventory (MBI). Covariates included gender, age, tenure, practice setting, employment status, family structure, and workplace support., | Overall prevalence was 42.9% for work-life integration (W: 34.3% vs. M: 55.4%), 77.8% for career satisfaction (W: 71.6% vs. M: 86.1%), and 44.5% for burnout (W: 53.7% vs. M: 33.7%); all gender differences were significant (p<0.01). Key correlates included structural factors (staffing, resources, scheduling), psychosocial aspects (recognition, support), and sleep deficits. | Female PEM physicians reported significantly lower work-life integration and career satisfaction, concurrent with higher burnout rates compared to men. | Cross-sectional design precludes causal inference. High risk of selection bias due to convenience sampling (single listserv). Limited external validity/generalizability regarding the broader PEM workforce. |
| Sánchez Onrubia I. (2025) | To assess the job satisfaction, work-family balance, sleep quality and burnout in nursing staff in various work time configurations (T1: rotating 7 / 7 / 10 hrs; T2: 12 hrs  shifts) | A cross-sectional observational study | Emergency department nurses of a tertiary hospital (T1 = 74; T2=104) | Data were collected via a composite questionnaire comprising sociodemographic covariates and two instruments validated for the Spanish population: the Maslach Burnout Inventory (MBI) and the Pittsburgh Sleep Quality Index (PSQI). Additionally, Job Satisfaction (JS) and Work-Life Balance (WLB) were assessed using metrics adopted from prior research | Regarding schedule flexibility, 71.2% (T1) and 66.7% (T2) of participants expressed satisfaction. High burnout rates were 8.9% and 10.3%, respectively. PSQI scores worsened over time (T1: 7.66 ±3.82 vs. T2: 8.46 ±4.05). Significant differences in work-family reconciliation were observed between rotating shift types (p < 0.05). | Staff preferred 12-h shifts for their positive impact on work-life balance, with no detrimental effects observed on sleep or occupational well-being (burnout, job satisfaction) | Cross-sectional design precludes causal inference. Small sample size limits statistical power and generalizability. |
| Nowrouzi-Kia B. (2022) | To assess personal and organizational determinants of Quality of Work Life (QoWL) among paramedics in northern Ontario, specifically evaluating the impact of Community Paramedicine practice. | A cross-sectional observational study | Paramedics (N=197) | 23-item Work-Related Quality of Life or WRQoL scale comprises six dimensions: Job and Career Satisfaction (JCS),  Working Conditions (WCS), General Well-Being (GWB), Home-Work Interface (HWI), Stress at Work (SAW), Control at Work (CAW) | The linear regression model for QoWL was statistically significant (R^2^ = 0.183, p <0.001). Higher QoWL scores were associated with better self-perceived mental health (b = 0.145, p = 0.002) and community paramedicine participation (b = 0.180, p = 0.048), whereas the number of sick days showed a negative association (b = -0.137, p < 0.001). Additionally, female paramedics reported significantly higher Job and Career Satisfaction (JCS). | Elevated paramedic QoWL was positively associated with better self-rated mental health and participation in Community Paramedicine (CP), while showing an inverse relationship with the frequency of sick leave. | Cross-sectional design precludes causal inference. Marginally low response rate (22.0%), introducing substantial non-response bias and severely compromising sample representativeness. |
| Sonmez L. (2021) | To evaluate differences in anxiety, burnout, and job satisfaction among emergency physicians, stratified by sociodemographic (age, gender) and occupational characteristics (tenure, professional role, and practice setting). | A cross-sectional observational study | Emergency medicine doctors (N=141) | Data were collected using three validated instruments: the Maslach Burnout Inventory (MBI) for occupational burnout, the State-Trait Anxiety Inventory (STAI) for anxiety, and the Short Form Minnesota Satisfaction Questionnaire (MSQ) for job satisfaction among emergency physicians. | Participants showed high emotional exhaustion (EE), moderate depersonalisation (DP) and personal achievement (PA). Anxiety levels were mild to moderate. In regard to job satisfaction, internal and overall levels were high, while external satisfaction was low. Younger age and shorter length of service were significantly associated with adverse burnout outcomes (higher EE/DP, lower PA). | Emergency physicians exhibited high levels of occupational burnout and anxiety, alongside low job satisfaction. The younger and less experienced clinicians are more prone to burnout and depersonalization. | Cross-sectional design precludes causal inference. Small sample size severely limits statistical power. Single-profession cohort restricts the generalizability of findings across the multidisciplinary emergency care team. |
| Feeks C. (2020) | To assess burnout prevalence and risk factors among Pediatric Emergency Medicine fellows. | A cross-sectional observational study | Pediatric emergency medicine physicians (N=139) | The Maslach Burnout Inventory (MBI) tool, Burnout prevalence was calculated with 95% confidence intervals (Agresti-Coull method) | Response rate = 30%, Women reported significantly higher burnout than men (39.8% vs. 13%; p=0.002). Similarly, single (50%) and divorced (66.7%) fellows exhibited higher rates compared to married colleagues (27%; p=0.008).  Primary contributors to burnout included work environment (52.5%), academic demands (36%), scheduling (35.3%), and work-life balance (33.8%). Training year was not associated with. | Burnout was significantly more prevalent among women and single or divorced fellows. | Cross-sectional design precludes causal inference. Low response rate (30%) introduces a substantial risk of non-response bias, significantly compromising sample representativeness. |
| Song N. (2022) | Assessment of occupational stressors and coping strategies among outpatient and emergency nurses in 29 pediatric hospitals in China. | Quantitative, cross-sectional ( multicentre) | Emergency/outpatient nurses from pediatric  speciality hospitals (N=1457) | Online questionnaire, Work Stressor Scale for Nurses (nurses’ expectations, work–family conflicts, interpersonal relationships at work, the nature of nursing work, patient factors, workload), The SimplifiedCope Style Questionnaire | The mean positive coping score was 36.66 ± 6.16. Work stressors (including WFC) were positively associated with negative coping styles (p < 0.01). Multiple regression identified age, professional experience, department, employment status, and negative coping styles as significant predictors of occupational stress. | Negative coping styles were prevalent among participants and positively associated with occupational stress. Significant predictors of stress levels included employment status, age, professional experience, department, and maladaptive coping strategies. | Cross-sectional design precludes causal inference. Data collection during the COVID-19 outbreak introduces a significant period effect. Atypical, crisis-driven professional solidarity may have artificially enhanced group cohesion, severely limiting the generalizability of these findings to standard, non-crisis operational settings. |
| Tong L. (2024) | Investigating the impact of effort-reward imbalance (ERI) on health and the mediating role of work-family conflict (WFC). | Quantitative, cross-sectional (prospective, multicentre) | Emergency nurses from 30 hospitals, N= 1,540, | Online questionnaire, ERI scale, WFBRC-S (work-family conflict), SSD-CN (somatic symptoms). PROCESS macro tool for statistical analysis. | ERI reported by 80.5% of respondents. ERI strongly correlates with somatic symptoms (r = 0.547). WFC acts as a mediator between ERI and health outcomes (β = 0.554, p < 0.001). Intrinsic effort moderates relationship between ERI and WFC - intrinsic effort could regulate the predictive effect of ERI on WFC (β = −0.104, p < 0.001). | WFC mediated the significant association between ERI and adverse health outcomes in emergency nurses. Intrinsic effort moderated this relationship: moderate investment buffered ERI’s impact, whereas excessive effort exacerbated burnout risk. | Cross-sectional design precludes causal inference. Limited transferability and external validity; findings are heavily bound to a specific geographical and socio-cultural health system context, restricting broader generalizability. |
| Tan Y. (2025) | To examine a moderated mediation model where work–family conflict conditions the indirect effect of ERI on somatic symptoms and sleep disorders via emotional exhaustion | Quantitative, cross-sectional | Emergency nurses from 30 hospitals, N= 1,540, | Online questionnaire, ERI scale, Maslach Burnout Inventory-General Survey (MBI-GS), Self-administered Sleep Questionnaire (SSQ), Somatization Symptom Self-Rating Scale-China (SSS-CN), Work-Family Behavioral Role Conflict Scale (WFBRC-S), PROCESS macro tool and Bootstrap procedure for statistical analysis. | The direct effect of ERI on somatic symptoms (β = 0.271) and sleep disorders (β = 0.137) remained significant (p < 0.01) after controlling for the mediator. Simple slope analysis indicated that low work–family conflict strengthened the ERI–emotional exhaustion relationship (slopes: 0.479 and 0.757, p < 0.01). The indices of moderated mediation were significant for both outcomes: −0.063 (95% CI: −0.077, −0.050) and −0.044 (95% CI: −0.056, −0.033). | WFC mediated the ERI–adverse health outcomes relationship. Intrinsic effort moderated this effect: moderate investment buffered negative impacts, whereas excessive effort exacerbated burnout risk. | Cross-sectional design precludes causal inference. Restricted generalizability due to a highly specific regional and socio-cultural context. |
| Williamson K. (2018) | To assess construct validity, correlations between MBI scores and established well-being instruments on the group of emergency medicine residents were analyzed. | Quantitative, cross-sectional (prospective, multicentre | Emergency medicine residents (N= 261) | The Maslach Burnout Inventory (MBI), The Quality Of Life (QoL) assessment with VAS scale, the Primary Care Evaluation of Mental Disorders Patient Health Questionnaire 2 question screen (PRIME-MD PHQ-2); Career satisfaction was assessed by the question: ‘‘If given the opportunity to revisit your career choice, would you choose to become a physician again?’’; Work-life balance was assessed with the question, ‘‘Does my work schedule leave me enough time for my personal/family life?’’ | Median Quality of Life (QoL) VAS score was 75 (IQR: 60–83.75). QoL correlated negatively with emotional exhaustion (r = -0.44) and depersonalization (r = -0.18), but positively with personal accomplishment (r = 0.35; all p < 0.01). While 52% reported positive work-life balance and 84% positive career satisfaction, both factors were associated with lower emotional exhaustion and depersonalization. A positive depression screen (40% prevalence) correlated with adverse scores across all MBI domains. Overall, 18% of residents met the criteria for burnout | Reduced quality of life, negative work-life balance, diminished career satisfaction, and positive depression screening (PHQ-2) were associated with elevated burnout severity. | Cross-sectional design precludes causal inference. Single-specialty cohort restricted exclusively to emergency medicine residents. |
| Osmančević B. (2023) | To investigate the perceptions of Emergency Medical Services (EMS) personnel regarding their occupational physical and mental well-being. | The qualitative study | The study sample comprised 26 EMS team members, including physicians (N=9), nurses (N=11), and ambulance drivers (N=6). | This qualitative study, grounded in Whitehead and Dahlgren’s framework, was conducted at the Teaching Institute for Emergency Medicine of the County of Istria, Croatia. Data saturation was achieved after 22 interviews. Subsequently, data were analyzed using a thematic analysis approach. In accordance with Steen and Roberts, inductive coding was employed, allowing themes to emerge directly from the data. | Physical: Predominance of musculoskeletal disorders (spine, joints) and cardiovascular issues, particularly among older personnel. Mental: Chronic stress (key stressors: pediatric cases, death, unpredictability) leading to burnout and emotional blunting. Sleep: Circadian rhythm disruption necessitates the use of sleep medication (sedatives/hypnotics), even among the youngest staff members (aged 25–26). Diet & Exercise: Meals are irregular and of low quality (characterized as "opportunistic eating"). Post-shift fatigue hinders adherence to physical training regimes despite high motivation. Substances: Tobacco smoking is adopted as a stress-reduction coping mechanism, even among previously non-smoking individuals. Spillover effect: The transfer of work-related tension into domestic relationships and family life. Identity: Difficulty distinguishing the professional role from private life (the "paramedic 24/7" mentality). Safety: The team serves as a trusted support system, whereas patients and the intervention environment are perceived as sources of threat and aggression. | Prolonged service in Emergency Medical Services (EMS) can have detrimental effects on employee health and job satisfaction. Consequently, preventive strategies—such as health promotion programs, active employee feedback mechanisms, and targeted training—are crucial for safeguarding occupational well-being. | Single-center design severely restricts external validity and broad generalizability. Limited transferability; findings are deeply rooted in the specific socio-cultural and regional health system context of Istria, Croatia. |
